# Surgical Margin Status and Minimal Margin Width in Penile Squamous Cell Carcinoma: Local Recurrence and Survival Outcomes in a Single-Centre Cohort

**DOI:** 10.3390/cancers18101535

**Published:** 2026-05-09

**Authors:** Mateusz Czajkowski, Michał Falis, Jan Mandrysz, Magdalena Sternau, Marcin Matuszewski, Oliver W. Hakenberg

**Affiliations:** 1 Department of Urology, Medical University of Gdańsk, Mariana Smoluchowskiego 17 Street, 80-214 Gdańsk, Poland; msternau@gumed.edu.pl (M.S.); marcin.matuszewski@gumed.edu.pl (M.M.); 2 Penile Disease Center, University Clinical Center of Gdańsk, Mariana Smoluchowskiego 17 Street, 80-214 Gdańsk, Poland; 3 Student Scientific Circle at the Department of Urology, Faculty of Medicine, Medical University of Gdańsk, Marii Skłodowskiej-Curie 3a, 80-210 Gdańsk, Poland; m.falis@gumed.edu.pl; 4 Division of Radiology Informatics and Statistics, Medical University of Gdańsk, Tuwima 15 Street, 80-210 Gdańsk, Poland; jan.mandrysz@gumed.edu.pl; 5 Department of Urology, Jena University Hospital, 07747 Jena, Germany; oliver.hakenberg@med.uni-jena.de

**Keywords:** penile cancer, squamous cell carcinoma, surgical margins, organ-sparing surgery, local recurrence, cancer-specific survival

## Abstract

**Background/Objectives**: Optimal surgical margin management in penile squamous cell carcinoma remains debated because organ-preserving surgery must balance oncological control with functional preservation. Historically, wide excision margins have been recommended; however, subsequent evidence has challenged this threshold, shifting practice towards narrower margins without a demonstrated increase in local recurrence. We evaluated whether invasive positive surgical margins and minimal negative margin widths were associated with local recurrence and survival after surgery for penile squamous cell carcinoma. **Methods**: We retrospectively analysed 157 consecutive men who underwent surgical treatment at a single centre between 2011 and 2024. Time-to-event analyses were performed in 131 patients with invasive non-metastatic disease after excluding those with penile intraepithelial neoplasia (PeIN)-only lesions (*n* = 23) and distant metastases (*n* = 3) at diagnosis. The margins were classified as either invasive-negative or invasive-positive. Among histologically negative-margin cases, minimal margin width was grouped a priori as <2 mm, 2–5 mm, and >5 mm. **Results**: The median follow-up was 25 months (interquartile range [IQR], 10–52). In the invasive (M0) cohort, 101/131 patients had invasive-negative margins and 30/131 had invasive-positive margins; local recurrence occurred in 42/131 patients. Margin status was not independently associated with recurrence-free, overall, or cancer-specific survival rates. Non-sparing surgery was associated with a lower hazard of local recurrence, whereas grade 3 (G3) histology independently predicted worse recurrence-free, overall, and cancer-specific survival. Advanced stage according to the Tumour, Node, Metastasis (TNM) classification independently predicted worse cancer-specific survival. **Conclusions**: Among patients with histologically negative margins, outcomes did not differ significantly across the predefined margin-width categories. These findings support tissue-preserving surgery aimed at histologically negative margins within a structured surveillance framework.

## Simple Summary

This retrospective single-centre cohort study addresses a clinically relevant question in penile cancer surgery: whether invasive positive surgical margins and minimal negative margin width are associated with local recurrence and survival after surgical treatment of penile squamous cell carcinoma. Organ-preserving surgery must balance oncological control with preservation of urinary and sexual function; however, the minimum safe resection margin remains debated. In an analytic cohort of 131 patients with invasive non-metastatic disease treated between 2011 and 2024, invasive positive margins were not independently associated with recurrence-free, overall, or cancer-specific survival after adjustment, whereas tumour grade and stage showed more consistent prognostic associations. Among patients with histologically negative margins, no statistically significant prognostic threshold for minimal margin width was identified. These findings may inform surgical decision-making and contribute to the ongoing refinement of margin management strategies for penile cancer.

## 1. Introduction

Penile cancer (PeCa) is a rare malignancy that shows geographic variation with industrialisation [1]. Its incidence is 0.94 per 100,000 men in Europe and 0.5 per 100,000 in the US, comprising <1% of male malignancies [2,3]. However, in some parts of Africa, South America, and Asia, PeCa accounts for up to 10% of all male cancers [4,5]. The most important risk factors for PeCa include phimosis, lack of circumcision, smoking, HPV infection, obesity, and rural residence [6]. Despite some modifiable factors, the incidence is rising, particularly among younger populations [7]. GLOBOCAN projections indicate a potential 56% increase in PeCa incidence by 2040 [8]. Poland has shown increased mortality rates, likely due to delayed diagnosis and its 0.11% circumcision rate, leading to a higher prevalence of phimosis [9,10].

Prevention and stage-specific treatment are essential for managing PeCa. The EAU guidelines emphasise complete tumour removal with organ preservation whenever oncologically feasible, because the penis has important urinary, sexual, and psychosocial functions [11]. Early stage tumours can often be treated with organ-sparing surgery [12]. Although these approaches achieve survival outcomes comparable to penectomy, they are associated with a higher risk of local recurrence [11,12]. Surgical margin width affects penile function and the risk of recurrence. Traditional recommendations require 2 cm of tumour-free margins [13]. However, recent techniques suggest 1 mm margins for low-grade (G1) and 5 mm for high-grade (G3) tumours [14], with current EAU guidelines considering margins >1 mm adequate in smaller, less aggressive PeCa tumours [15]. Intraoperative frozen section margin assessment is not recommended because of its unreliability [16].

Organ-sparing surgery for PeCa increases the risk of positive surgical margins (PSMs), with conflicting evidence on its prognostic significance due to variable cohorts and definitions. Roussel et al. found that margin positivity correlated with higher local recurrence rates, although this lost significance when considering tumour stage and grade [16]. Jazayeri et al. showed that penile intraepithelial neoplasia (PeIN) at the margin did not increase the risk of recurrence and could be managed with surveillance [17]. While data exist on the minimal safe margins for pT1-pT2 PeCa, comprehensive analyses of the prognostic role of positive margins across all stages are lacking.

This study evaluated whether invasive positive surgical margins and minimal negative margin widths are associated with local recurrence, overall survival, and cancer-specific survival in an invasive non-metastatic cohort after dedicated uropathology re-review. The novelty of this analysis lies in the combined assessment of invasive margin positivity and measured minimal negative margin width in a consecutive real-world surgical cohort while separating invasive carcinoma at the inked margin from PeIN-only margin involvement. These data may help refine clinical recommendations from fixed wide-margin thresholds toward histologically negative, risk-adapted organ preservation with structured surveillance and timely salvage therapy.

## 2. Materials and Methods

This retrospective study included 157 consecutive patients treated at a tertiary centre between January 2011 and October 2024, in accordance with the STROBE guidelines. The study was approved by the Bioethics Committee for Scientific Research at the Medical University of Gdańsk (Decision No. NKBBN/168/2023) and was conducted in compliance with relevant institutional requirements and the Declaration of Helsinki. Informed consent was obtained from all the eligible patients. Eligible patients were aged ≥18 years and had undergone surgery for penile cancer after providing informed consent. Demographic and histopathological data were collected according to the 2020 WHO Classification and the 8th edition of the Tumour, Node, Metastasis (TNM) Classification [18,19]. The specimens were re-evaluated by a uropathologist between December 2024 and January 2025. Patients were classified as pNx when pathological nodal assessment was not performed because of low-risk disease managed with nodal surveillance. The preoperative regional lymph node status was recorded from physical examination and available imaging before primary surgery and classified as cN0, cN1, cN2, or cN3 according to the 8th edition of the TNM classification. This cohort partially overlapped with that of our previous study describing the clinical characteristics and overall prognostic profile of patients with penile cancer treated at our centre [6]. However, the present analysis addresses a distinct research question and reports analyses not presented previously, namely a dedicated uropathology re-review of invasive margin status and minimal negative margin width and their association with recurrence-free, overall, and cancer-specific survival in the invasive M0 cohort. Margins were classified as invasive-negative (no invasive carcinoma at the inked margin) or invasive-positive (invasive carcinoma at the inked margin; PSM). Among cases with invasive-negative margins, the minimal negative margin width was defined as the shortest distance between invasive carcinoma and the inked resection margin and categorised a priori as <2 mm, 2–5 mm, or >5 mm. These thresholds were not selected using data-driven cut-point optimisation. They were chosen to separate very close histological margins (<2 mm), contemporary narrow margins compatible with tissue-preserving surgery (2–5 mm), and wider negative margins (>5 mm). In particular, the EAU-ASCO guidelines summarise that most penile SCC lesions do not extend more than 5 mm beyond the macroscopic margin, that 5–10 mm excision margins have been associated with acceptably low recurrence rates, and that local recurrence appears to increase substantially when the tumour-to-margin distance is <1 mm. Therefore, the <2 mm category was used as a pragmatic close-margin group in this retrospective dataset, whereas >5 mm served as a clinically interpretable wider-margin reference category [11,13,20]. The presence of PeIN at the margin was recorded separately and was not classified as an invasive PSM unless an invasive carcinoma was present at an inked margin. Local recurrence was defined as histologically confirmed SCC at the primary surgical site after initial resection. Of the 157 surgically treated patients, 23 had PeIN/pTis without invasive carcinoma, and three presented with distant metastases at the time of diagnosis (cM1). These 26 patients were excluded from all margin status and time-to-event analyses, resulting in an invasive non-metastatic (M0) analytic cohort of 131 patients for RFS, OS, and CSS. The analyses of the minimal negative margin width were restricted to the R0 subgroup (*n* = 101). Postoperative management was performed according to the standard institutional protocol. Patients with positive margins underwent surveillance, and salvage surgery was performed only in cases of confirmed, local recurrence. When feasible, salvage treatment involved organ-sparing procedures for patients who initially underwent penile-preserving surgery. None of the patients received adjuvant radiotherapy before surgery.

Follow-up visits were conducted at 2 weeks for wound assessment and at one and three months. Patients were examined every three months for two years. After year three, the follow-up frequency was adapted to the individual risk according to the EAU guidelines [15].

## 3. Statistical Methods

Descriptive statistics were reported for the overall cohort (*n* = 157). All time-to-event analyses (RFS, OS, and CSS), including Kaplan–Meier estimates and Cox regression, were performed in the invasive M0 analytic cohort (*n* = 131), unless specified otherwise. Overall survival (OS) was defined as the time from surgery to death from any cause, with patients who were alive at the last follow-up being censored. Cancer-specific survival (CSS) was defined as the time from surgery to death from penile cancer, with non-cancer deaths being censored. Recurrence-free survival (RFS) was defined as the time to the first local recurrence at or near the surgical site after the initial resection. The minimal negative margin width was analysed only in the R0 subgroup (*n* = 101) and grouped as <2 mm, 2–5 mm, or >5 mm. Analyses were performed using R software (version 4.3.2). Variables are presented as medians with interquartile ranges (IQRs) for continuous data and frequencies with percentages for categorical data. Group comparisons were performed using the Mann–Whitney U or Kruskal–Wallis tests for continuous variables and the chi-square or Fisher’s exact test for categorical variables. Survival outcomes were estimated using the Kaplan–Meier method and compared using the log-rank test. Cox regression was used to evaluate the association between predictors and outcomes. For RFS and OS, the univariable and multivariable Cox models included surgery type, age at surgery, margin status, histological grade (G2 vs. G1; G3 vs. G1), TNM stage (pT2 N0-Nx vs. pT1 N0-Nx; N+ and/or ≥pT3 vs. pT1 N0-Nx), and p16 status. For CSS, the same covariates were used, except for p16 status, which was excluded because no cancer-specific deaths occurred in the p16-positive subgroup. The results are reported as hazard ratios (HRs) with 95% confidence intervals (CIs). No formal a priori sample size or power calculations were performed. The sample size was determined by the number of consecutive surgically treated patients with penile squamous cell carcinoma available during the predefined study period and by applying predefined clinical inclusion and exclusion criteria. Given the retrospective design and rarity of penile cancer, the analyses were considered exploratory and interpreted using effect estimates, 95% confidence intervals, and *p*-values. Subgroup analyses were interpreted cautiously because of the potential risk of limited statistical power in the analyses. Statistical significance was set at *p* < 0.05.

## 4. Results

### 4.1. Patient and Tumour Characteristics

**Figure 1:**
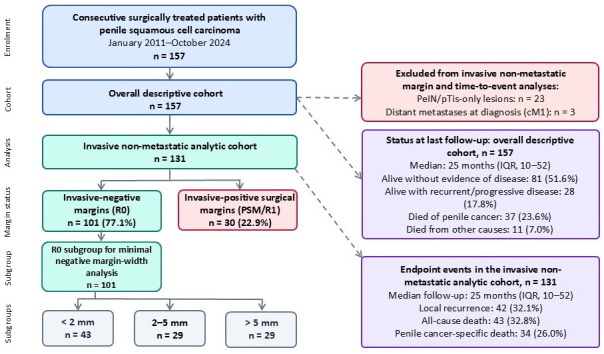
Patient-flow diagram. The overall descriptive cohort included 157 consecutive patients who underwent surgery for penile squamous cell carcinoma between January 2011 and October 2024. PeIN/pTis-only lesions (*n* = 23) and patients with distant metastases at diagnosis (cM1; *n* = 3) were excluded from the invasive non-metastatic analytic cohort, resulting in 131 patients for margin and time-to-event analyses. Minimal negative margin-width analyses were restricted to patients with invasive-negative margins (R0, *n* = 101). PeIN, penile intraepithelial neoplasia; PSM, positive surgical margin.

The overall cohort comprised 157 patients treated surgically between January 2011 and October 2024, including 23 with high-grade PeIN/pTis and 134 with invasive tumours (≥pT1). The median follow-up (time from surgery to last contact or death) in the overall cohort was 25 months (interquartile range [IQR], 10–52; range, 9–161 months). For time-to-event analyses (RFS, OS, CSS), PeIN-only lesions (*n* = 23) and patients with distant metastases at diagnosis (cM1; *n* = 3) were excluded, resulting in an invasive M0 analytic cohort of 131 patients. The median age was 64 years (range, 30–87 years), and the median body mass index was 29 kg/m^2^ (range, 16–38 kg/m^2^). At the last follow-up, 81 patients (51.6%) were alive without evidence of disease, 28 (17.8%) were alive with recurrent/progressive disease, 37 (23.6%) had died of penile cancer, and 11 (7.0%) had died of other causes. The demographic and clinical data are summarised in Table 1 and Figure 1.

### 4.2. Surgical Margins

In the overall cohort, invasive-positive surgical margins (PSMs) were observed in 32/157 patients (20.4%). In the invasive M0 analytic cohort (*n* = 131), 101 patients (77.1%) had invasive-negative margins and 30 (22.9%) had invasive-positive margins. Among the 101 R0 patients, the minimal negative invasive margin width was <2 mm in 43 patients, 2–5 mm in 29 patients, and >5 mm in 29 patients; the median minimal negative invasive margin width was 2.8 mm (range, 0.1–20 mm). Invasive PSMs were more frequent in high-grade (G3) tumours than in low-grade (G1) tumours.

### 4.3. Local Recurrence and Recurrence-Free Survival

**Figure 2:**
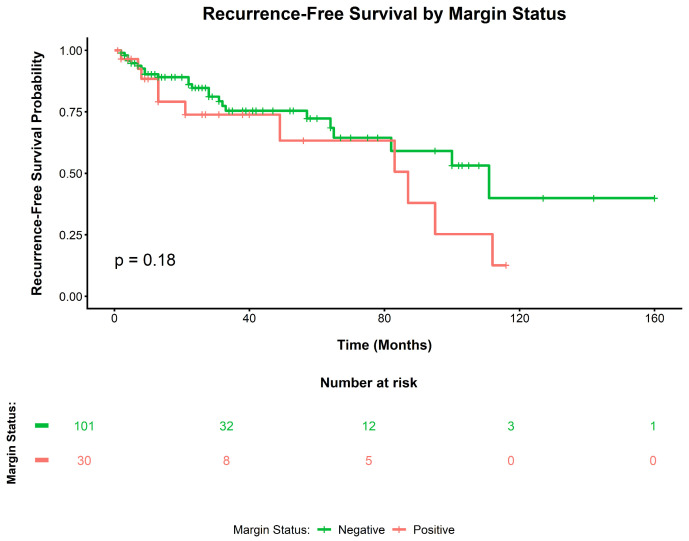
Kaplan–Meier curves for local recurrence-free survival (RFS) according to surgical margin status in the invasive M0 analytic cohort (negative *n* = 101; positive *n* = 30). No significant difference was observed between the groups (log-rank, *p* = 0.18).

**Figure 3:**
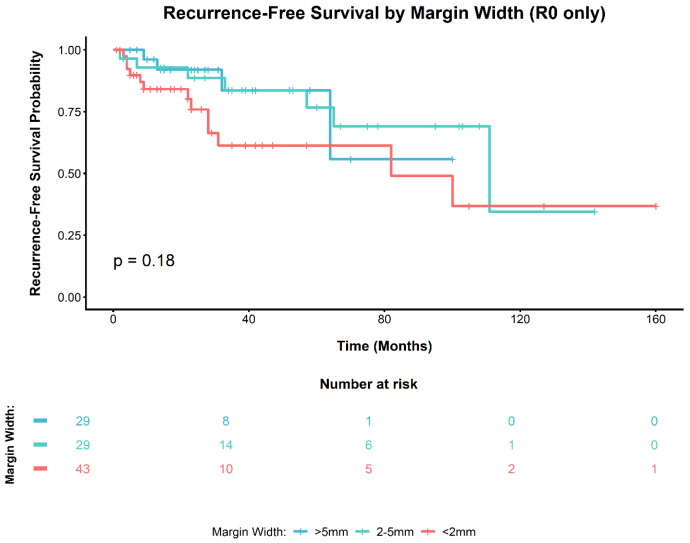
Kaplan–Meier curves for local recurrence-free survival (RFS) according to minimal negative margin width (<2 mm, 2–5 mm, and >5 mm) among R0 patients in the invasive M0 analytic cohort (*n* = 101). No significant differences were observed across the predefined margin-width categories (log-rank *p* = 0.18).

During follow-up, local recurrence occurred in 42/157 patients (26.8%). All local recurrences occurred in the invasive M0 analytic cohort (42/131, 32.1%). The Kaplan–Meier curves for recurrence-free survival according to the invasive margin status in the invasive M0 analytic cohort are shown in Figure 2 and did not demonstrate a significant between-group difference (log-rank *p* = 0.18). Among R0 patients (*n* = 101), RFS did not differ significantly across the minimal negative margin-width categories (<2 mm, *n* = 43; 2–5 mm, *n* = 29; >5 mm, *n* = 29; log-rank *p* = 0.18; Figure 3). In the invasive non-metastatic analytic cohort (*n* = 131), 18 patients (13.7%) were HPV-positive and 113 (86.3%) were HPV-negative. In the invasive non-metastatic analytic cohort, local recurrence occurred in 8 of 18 HPV-positive patients (44.4%) and in 34 of 113 HPV-negative patients (30.1%). However, this difference was not statistically significant (Fisher’s exact test, *p* = 0.568).

In the univariable Cox regression analysis of the invasive M0 analytic cohort (*n* = 131), non-sparing surgery showed a borderline association with a lower hazard of local recurrence than sparing surgery (HR 0.54, 95% CI 0.26–1.12; *p* = 0.096), whereas G3 histology (vs. G1) was associated with worse RFS (HR 3.45, 95% CI 1.21–9.81; *p* = 0.020). Margin positivity was not significantly associated with local recurrence (HR 1.64, 95% CI 0.80–3.36; *p* = 0.179). In the multivariable Cox regression, non-sparing surgery remained associated with a lower hazard of local recurrence (adjusted HR 0.41, 95% CI 0.19–0.89; *p* = 0.024), and G3 histology independently predicted worse RFS (adjusted HR 4.98, 95% CI 1.58–15.72; *p* = 0.006). Margin status remained non-significant (adjusted HR 1.90, 95% CI 0.90–4.04; *p* = 0.094), while age, G2 histology, TNM stage, and HPV/p16 status were not independently associated with RFS (Table 2; Figure S1).

### 4.4. Overall Survival and Prognostic Factors

At the time of analysis, 48/157 patients (30.6%) had died in the overall cohort. Overall survival (OS) analyses were performed in the invasive M0 analytic cohort (*n* = 131; 43 deaths). Kaplan–Meier curves showed no significant differences in OS according to margin status (log-rank *p* = 0.83) or minimal margin width among R0 patients (log-rank *p* = 0.15), whereas surgery type showed a non-significant trend (log-rank *p* = 0.082) (Figures S2 and S3).

In the univariable Cox regression analysis, margin status was not associated with OS (HR 1.08, 95% CI 0.52–2.26; *p* = 0.833). G3 histology (vs. G1) and advanced TNM stage (N+ and/or ≥pT3 vs. pT1 N0–Nx) were associated with worse OS (HR 5.22, 95% CI 2.09–13.07; *p* < 0.001 and HR 3.09, 95% CI 1.40–6.82; *p* = 0.005, respectively), whereas HPV-positive status showed a non-significant trend towards improved OS (HR 0.15, 95% CI 0.02–1.08; *p* = 0.060). In the multivariable Cox regression, margin status remained non-significant (adjusted HR 0.89, 95% CI 0.42–1.90; *p* = 0.763). G3 histology independently predicted worse OS (adjusted HR 3.15, 95% CI 1.14–8.73; *p* = 0.028), while advanced TNM stage showed a borderline association (adjusted HR 2.27, 95% CI 0.95–5.45; *p* = 0.066). Surgery type, age, G2 histology, pT2 stage, and HPV status were not independently associated with OS (Table 3; Figure S4).

### 4.5. Cancer-Specific Survival and Prognostic Factors

In the overall cohort, 37/157 patients (23.6%) died of penile cancer. Cancer-specific survival (CSS) analyses were performed in the invasive M0 analytic cohort (*n* = 131; 34 deaths due to penile cancer). Kaplan–Meier curves showed no significant differences in CSS according to margin status (log-rank *p* = 0.96) or minimal margin width among R0 patients (log-rank *p* = 0.11), whereas non-sparing surgery was associated with worse CSS in the unadjusted analysis (log-rank *p* = 0.034).

In the univariable Cox regression analysis, margin status was not associated with CSS (HR 1.02, 95% CI 0.44–2.34; *p* = 0.966). Non-sparing surgery (HR 2.12, 95% CI 1.04–4.33; *p* = 0.038), G3 histology (HR 10.37, 95% CI 2.99–36.03; *p* < 0.001), and advanced TNM stage (N+ and/or ≥pT3 vs. pT1 N0-Nx: HR 5.34, 95% CI 1.86–15.36; *p* = 0.002) were associated with worse CSS in the univariable analysis. In the multivariable model, margin status remained non-significant (adjusted HR 0.79, 95% CI 0.34–1.85; *p* = 0.586), whereas G3 histology (adjusted HR 4.71, 95% CI 1.21–18.30; *p* = 0.025) and advanced TNM stage (adjusted HR 3.54, 95% CI 1.12–11.18; *p* = 0.031) independently predicted worse CSS. Non-sparing surgery showed a borderline association after adjustment (adjusted HR 2.03, 95% CI 0.97–4.26, *p* = 0.060). HPV/p16 status was excluded from CSS modelling because no cancer-specific deaths occurred in the HPV-positive subgroup (Table 4, Figure S5).

### 4.6. Management of Local Recurrence

Of 42 local recurrences, 39 occurred after primary organ-sparing surgery. Salvage treatment included penile-preserving procedures in 36 patients (92.3%) and partial penectomy in three patients (7.7%). For three recurrences after non-organ-sparing surgery, two patients underwent extended partial penectomy, and one underwent total penectomy.

## 5. Discussion

In this high-volume, single-centre cohort, invasive positive surgical margins were not independently associated with RFS, OS, or CSS. For local control, non-sparing surgery was associated with a lower hazard of local recurrence than sparing surgery, whereas G3 histology independently predicted worse RFS. For the survival endpoints analysed in the invasive M0 cohort (*n* = 131), G3 histology independently predicted both OS and CSS, and advanced TNM stage independently predicted CSS while showing a borderline association with OS. Among patients with negative margins, no significant differences in OS or CSS were observed across the predefined negative margin-width categories. At our centre, patients with positive margins were managed using a surveillance-first strategy without adjuvant radiotherapy, with salvage surgery reserved for histologically confirmed local recurrence. Moreover, most recurrences after organ-sparing surgery were amenable to repeated penile-preserving procedures.

Across contemporary penile-sparing and amputative series, PSM rates typically range from 10% to 16% and are generally associated with increased local recurrence, while overall survival remains unchanged. In addition, routine intraoperative margin assessment does not eliminate local relapse, and cancer-related deaths are largely concentrated in node-positive disease [21,22,23,24]. Our PSM rate was slightly higher (22.9% in the invasive M0 cohort), which likely reflects the high utilization of organ-sparing procedures in an all-stage cohort and an intentional preference for preservation with structured surveillance. Consistent with previous studies, our data support the concept that PSMs do not necessarily translate into worse long-term survival.

Recommended surgical margins in penile cancer have narrowed from the historical 2-cm dogma to millimetre-scale targets, supported by pathological studies showing limited microscopic extension beyond the apparent tumour [25,26,27]. In our cohort, among patients with histologically negative margins, we found no significant difference in recurrence-free survival across the predefined minimal margin-width categories (<2, 2–5, and >5 mm; log-rank *p* = 0.18). Likewise, no significant differences were observed in OS or CSS according to the margin width category. In summary, the data did not indicate a definitive clinically relevant prognostic threshold within histologically negative margins in this cohort. Instead, they support a strategy that prioritises microscopically negative resection while maximising tissue preservation.

Several studies suggest that local recurrence after penile-sparing surgery is driven more by tumour biology than by small differences in negative margin width and that local relapse does not necessarily translate into worse cancer-specific survival when timely salvage is feasible [28,29]. However, the organ preservation is not only cosmetic or anatomical; it directly affects urinary and sexual outcomes. In the comparative series by Cilio et al., patients with localised glans-confined penile cancer treated with wide local excision had better postoperative sexual-function outcomes than those treated with glansectomy with urethral glanduloplasty; glansectomy was associated with erectile dysfunction and lower Changes in Sexual Function Questionnaire scores [30]. These data support use of the least morbid oncologically adequate organ-sparing procedure and underline the importance of defining safe histological margin policies. In our cohort, G3 histology independently predicted worse RFS, whereas survival outcomes were driven mainly by grade and stage. Most local recurrences after organ-sparing surgery were managed surgically within a structured follow-up pathway. HPV/p16 status was not significantly associated with local recurrence (44.4% vs. 30.1%; Fisher’s exact test, *p* = 0.568), but this analysis was underpowered because the HPV/p16-positive subgroup was small. Our results are consistent with the view that adverse tumour biology, rather than minor differences in negative margin width, is the dominant determinant of recurrence and survival in penile SCC. High grade, advanced local stage, and adverse pathological features consistently predict relapse and cancer-specific mortality [31]. Positive invasive margins may contribute to local failure, particularly in more advanced disease, whereas isolated PeIN at the margin appears to behave differently and can often be managed with surveillance [16,17]. In our multivariable analysis, G3 histology remained an independent predictor of local recurrence, while invasive margin positivity showed only a borderline, non-significant association with RFS. Survival was driven predominantly by grade and stage.

Our findings contribute to the understanding of local recurrence after organ-sparing surgery. In our R0 subgroup, we also found no significant difference in RFS across the predefined minimal negative margin width categories. Elst et al. reported 29% local recurrence in 550 mostly pT1 tumours after preservation procedures, with 99% 5-year cancer-specific survival [32]. PeIN at the margin has been associated with a higher risk of local relapse, suggesting that at least some recurrences may represent new tumours arising from dysplastic epithelium [17]. Roussel et al.’s analysis of 897 pT2-3 tumours showed high-grade histology and pT3 stage predicted local recurrence and worse survival [16]. In our cohort, margin positivity was not significantly associated with local recurrence (univariable HR 1.64, 95% CI 0.80–3.36; adjusted HR 1.90, 95% CI 0.90–4.04), whereas non-sparing surgery was associated with a lower adjusted hazard of local recurrence (adjusted HR 0.41, 95% CI 0.19–0.89), and G3 histology independently predicted recurrence (adjusted HR 4.98, 95% CI 1.58–15.72). Similarly, OS and CSS were not associated with margin status but were influenced by tumour grade and TNM stage. These findings advocate for organ-sparing surgery with subsequent follow-up, considering pathological characteristics and nodal risk when making decisions regarding secondary surgical interventions.

Data on whether HPV-associated penile SCC requires different margin policies are limited, and the available evidence does not support tailoring margin width based on HPV/p16 status [17,33,34]. In our cohort, HPV/p16 status was not associated with RFS or OS and could not be modelled for CSS because no cancer-specific deaths occurred in the HPV-positive subgroup. These findings, while underpowered, support the view that established pathological factors, rather than HPV status itself, remain the main determinants of prognosis.

From a clinical perspective, these findings support a surveillance-first approach for selected patients with invasive PSMs, provided that the follow-up is structured and sufficiently intensive to detect early local relapse. In our institutional protocol, salvage surgery was reserved for histologically confirmed local recurrence and was frequently feasible with penile-preserving techniques after organ-sparing primary treatment. This strategy may avoid immediate re-resection in every case while maintaining oncological safety at the level of survival.

This study had several strengths and limitations that should be considered. Strengths include a large consecutive cohort from a national tertiary centre, a 13-year observation period, and incorporation of all pathological stages and treatments, facilitating the evaluation of margin status in a real-world context. All specimens were re-examined by an experienced uropathologist, and outcomes were analysed using Cox models in the invasive M0 analytic cohort after excluding PeIN-only lesions and cM1 disease. For RFS and OS, the multivariable models included surgery type, age, margin status, histological grade, TNM stage categories, and HPV/p16 status. For CSS, the same covariates were used, except for HPV/p16, which was omitted because no cancer-specific deaths occurred in the HPV-positive subgroup. Limitations include its retrospective single-centre design, potential selection bias, limited power in margin-width subgroups, and absence of patient-reported functional outcomes. Due to the retrospective design and the rarity of penile cancer, no a priori sample-size or power calculation was performed. The sample size was determined by the number of consecutive patients meeting the predefined inclusion and exclusion criteria during the study period. Therefore, some subgroup analyses may have been underpowered to detect smaller but clinically meaningful differences, and a risk of Type II error cannot be excluded. Additionally, pathological nodal staging was incomplete: 36 patients (22.9%) were pNx because pathological nodal assessment was not performed, generally in clinically lower-risk patients managed with nodal surveillance. As pNx status was therefore not missing at random, occult nodal disease in this subgroup could have misclassified some patients into the lower-stage reference categories and would most likely attenuate the observed effect of nodal stage on survival. Moreover, the cohort included heterogeneous procedures, ranging from circumcision and wide local excision to total penectomy, over more than a decade. Although surgery type was included in multivariable models as organ-sparing versus non-sparing surgery, this adjustment cannot fully account for treatment indication, tumour location and extent, surgeon preference, increasing institutional experience, referral centralisation, or changes in follow-up intensity over time. Prospective randomized multicentre studies integrating standardised nodal management, patient-reported outcomes, and molecular markers are recommended. Finally, the study period spanned more than a decade, during which institutional experience and organisational structures may have evolved (including the centralisation of PeCa referrals). Such time trends could have influenced the case mix, surgical technique, and follow-up intensity and should be considered when interpreting long-term outcomes.

## 6. Conclusions

In this retrospective single-centre cohort, invasive positive surgical margins were not independently associated with local recurrence, overall survival, or cancer-specific survival after adjustment. Tumour grade and stage showed more consistent prognostic associations: G3 histology independently predicted worse RFS, OS, and CSS, and advanced TNM stage independently predicted worse CSS. Among R0 patients, no statistically significant prognostic threshold for minimal negative margin width was identified. These findings support a tissue-preserving strategy aimed at histologically negative margins within a structured surveillance-and-salvage pathway. They should not be interpreted as evidence of equivalence because this study was retrospective and limited by incomplete pathological nodal staging, procedural heterogeneity, and limited subgroup power. Future recommendations should be informed by prospective multicentre datasets and, where feasible, randomised or pragmatic comparative studies incorporating standardised nodal staging, margin reporting, and functional patient-reported outcomes.

## Figures and Tables

**Table 1:** Baseline demographic, clinicopathological, and treatment characteristics of 157 patients with penile squamous cell carcinoma.

Variable	*n* (%) or Median (Range)
age at surgery (years)	64 (30–87)
body mass index (BMI, kg/m^2^)	29 (16–38)
follow-up (months)	25 (IQR 10–52; 9–161)
**survival status**	
Alive	109 (69.4%)
– alive without disease	81 (51.6%)
– alive with disease (recurrence/progression)	28 (17.8%)
Deceased	48 (30.6%)
– due to penile cancer	37 (77.1%)
– due to other causes *	11 (22.9%)
**tumour type**	
Usual-type squamous cell carcinoma	157 (100%)
**histologic grade**	
high-grade PeIN (ca in situ)	23 (14.6%)
G1 (well differentiated)	35 (22.3%)
G2 (moderately differentiated)	71 (45.2%)
G3 (poorly differentiated)	28 (17.8%)
**HPV/p16 status (overall cohort)**	
Positive	24 (15.3%)
Negative	133 (84.7%)
**HPV/p16 status (invasive non-metastatic M0)**	
Positive	18 (13.7%)
Negative	113 (86.3%)
**clinical stage (cT)**	
cTis	25 (15.9%)
cT1	58 (36.9%)
cT2	42 (26.8%)
cT3	27 (17.2%)
cT4	5 (3.2%)
**pathological stage (pT)**	
PeIN (high-grade)	23 (14.6%)
pT1a	44 (28.0%)
pT1b	20 (12.7%)
pT2	41 (26.1%)
pT3	24 (15.3%)
pT4	5 (3.2%)
**clinical nodal status (cN)**	
cN0	110 (70.1%)
cN1	17 (10.8%)
cN2	12 (7.6%)
cN3	18 (11.5%)
**pathological nodal status (pN)**	
pN0	71 (45.2%)
pN1	19 (12.1%)
pN2	10 (6.4%)
pN3	21 (13.4%)
pNx (not assessed) **	36 (22.9%)
**distant metastases (cM)**	
cM0	154 (98.1%)
cM1	3 (1.9%)
**type of primary surgery**	
Circumcision	20 (12.7%)
wide local excision	12 (7.6%)
glansectomy + skin graft	57 (36.3%)
partial penectomy + skin graft	51 (32.5%)
partial penectomy without graft	10 (6.4%)
Total penectomy	7 (4.5%)
**surgical margin status in the invasive non-metastatic M0 analytic cohort (*****n*** **= 131)**	
Negative	101 (77.1%)
Positive	30 (22.9%)
minimal negative margin width, among R0 cases (mm)	2.8 (0.1–20)

Data are presented as median (range) or numbers (percentages). Abbreviations: IQR, interquartile range; M0, no distant metastases; PeIN, penile intraepithelial neoplasia. * Other causes included cardiovascular disease (*n* = 3), secondary malignancies (*n* = 4; lung, rectal cancer, and multiple myeloma), cerebrovascular events (*n* = 2), liver failure (*n* = 1), and COVID-19 infection (*n* = 1). ** Pathological nodal staging was not performed in clinically cN0 patients with low-risk primary tumours (<pT1b), who were managed with nodal surveillance.

**Table 2:** Univariable and multivariable Cox proportional hazards regression analyses for local recurrence-free survival according to surgery type, age, surgical margin status, histological grade, TNM stage, and HPV/p16 status.

Parameter, 131 Patients, 42 Recurrences	Univariable *p*-Value	Univariable HR (95% CI)	Multivariable *p*-Value	Multivariable HR (95% CI)
Surgery (Non-sparing vs. Sparing)	0.096	0.54 (0.26–1.12)	0.024 *	0.41 (0.19–0.89)
Age at surgery (years)	0.990	1.00 (0.97–1.03)	0.815	1.00 (0.97–1.03)
Margin status (Positive vs. Negative)	0.179	1.64 (0.80–3.36)	0.094	1.90 (0.90–4.04)
Histological grade (G2 vs. G1)	0.163	1.92 (0.77–4.78)	0.130	2.07 (0.81–5.34)
Histological grade (G3 vs. G1)	0.020 *	3.45 (1.21–9.81)	0.006 **	4.98 (1.58–15.72)
TNM stage (pT2 N0-Nx vs. pT1 N0-Nx)	0.136	0.43 (0.14–1.30)	0.057	0.33 (0.11–1.03)
TNM stage (N+ and/or ≥pT3 vs. pT1 N0-Nx)	0.871	0.94 (0.46–1.92)	0.100	0.51 (0.23–1.14)
HPV/p16 status (Positive vs. Negative)	0.603	1.27 (0.52–3.07)	0.873	1.08 (0.44–2.66)

Significance: * *p* < 0.05; ** *p* < 0.01; HR, hazard ratio; CI, confidence interval.

**Table 3:** Univariable and multivariable Cox proportional hazards regression analyses for overall survival according to surgery type, age, surgical margin status, histological grade, TNM stage, and HPV/p16 status.

Parameter, 131 Patients, 43 Deaths	Univariable *p*-Value	Univariable HR (95% CI)	Multivariable *p*-Value	Multivariable HR (95% CI)
Surgery (Non-sparing vs. Sparing)	0.085	1.74 (0.93–3.28)	0.106	1.72 (0.89–3.30)
Age at surgery (years)	0.083	1.03 (1.00–1.05)	0.250	1.02 (0.99–1.05)
Margin status (Positive vs. Negative)	0.833	1.08 (0.52–2.26)	0.763	0.89 (0.42–1.90)
Histological grade (G2 vs. G1)	0.207	1.75 (0.73–4.16)	0.520	1.35 (0.54–3.37)
Histological grade (G3 vs. G1)	<0.001 ***	5.22 (2.09–13.07)	0.028 *	3.15 (1.14–8.73)
TNM stage (pT2 N0-Nx vs. pT1 N0-Nx)	0.679	1.24 (0.45–3.42)	0.974	1.02 (0.36–2.90)
TNM stage (N+ and/or ≥pT3 vs. pT1 N0-Nx)	0.005 **	3.09 (1.40–6.82)	0.066	2.27 (0.95–5.45)
HPV/p16 status (Positive vs. Negative)	0.060	0.15 (0.02–1.08)	0.074	0.16 (0.02–1.20)

Significance: * *p* < 0.05; ** *p* < 0.01; *** *p* < 0.001; HR = Hazard Ratio; CI = Confidence Interval.

**Table 4:** Univariable and multivariable Cox proportional hazards regression analyses for cancer-specific survival according to surgery type, age, surgical margin status, histological grade, and TNM stage.

Parameter, 131 Patients, 34 Penile Cancer Deaths	Univariable *p*-Value	Univariable HR (95% CI)	Multivariable *p*-Value	Multivariable HR (95% CI)
Surgery (Non-sparing vs. Sparing)	0.038 *	2.12 (1.04–4.33)	0.060	2.03 (0.97–4.26)
Age at surgery (years)	0.263	1.02 (0.99–1.05)	0.381	1.01 (0.98–1.05)
Margin status (Positive vs. Negative)	0.966	1.02 (0.44–2.34)	0.586	0.79 (0.34–1.85)
Histological grade (G2 vs. G1)	0.101	2.82 (0.82–9.75)	0.343	1.87 (0.51–6.84)
Histological grade (G3 vs. G1)	<0.001 ***	10.37 (2.99–36.03)	0.025 *	4.71 (1.21–18.30)
TNM stage (pT2 N0-Nx vs. pT1 N0-Nx)	0.431	1.70 (0.46–6.32)	0.584	1.46 (0.38–5.68)
TNM stage (N+ and/or ≥pT3 vs. pT1 N0-Nx)	0.002 **	5.34 (1.86–15.36)	0.031 *	3.54 (1.12–11.18)

Significance: * *p* < 0.05; ** *p* < 0.01; *** *p* < 0.001; HR = hazard ratio; CI = confidence interval.

## Data Availability

De-identified questionnaire data underlying the main analyses and analytic code can be made available upon reasonable request to the corresponding author, subject to institutional approval and signing of a data use agreement.

## Supplementary Materials

The following supporting information can be downloaded at: https://www.mdpi.com/article/10.3390/cancers18101535/s1, Figure S1: Forest plot of univariable and multivariable Cox proportional hazards models for recurrence-free survival (RFS) in the invasive M0 analytic cohort; Figure S2: Kaplan–Meier curves for overall survival (OS) according to invasive surgical margin status in the invasive M0 analytic cohort; Figure S3: Kaplan–Meier curves for overall survival (OS) according to minimal negative margin width (<2 mm, 2–5 mm, >5 mm) among R0 patients in the invasive M0 analytic cohort; Figure S4: Forest plot of univariable and multivariable Cox proportional hazards models for overall survival (OS) in the invasive M0 analytic cohort; Figure S5: Forest plot of univariable and multivariable Cox proportional hazards models for cancer-specific survival (CSS) in the invasive M0 analytic cohort.

## Abbreviations

BMI	body mass index
CI	confidence interval
CSS	cancer-specific survival
EAU	European Association of Urology
HR	hazard ratio
IQR	interquartile range
OS	overall survival
PeCa	penile cancer
PeIN	penile intraepithelial neoplasia
PSM	positive surgical margin
RFS	recurrence-free survival
STROBE	Strengthening the Reporting of Observational Studies in Epidemiology

## References

[1] Giona S., Barber N., Ali A. (2022). The Epidemiology of Penile Cancer. Urologic Cancers.

[2] Fu L., Tian T., Yao K., Chen X.-F., Luo G., Gao Y., Lin Y.-F., Wang B., Sun Y., Zheng W. (2022). Global Pattern and Trends in Penile Cancer Incidence: Population-Based Study. JMIR Public Health Surveill..

[3] Douglawi A., Masterson T.A. (2017). Updates on the Epidemiology and Risk Factors for Penile Cancer. Transl. Androl. Urol..

[4] Richter S., Ruether J.D., Wood L., Canil C., Moretto P., Venner P., Gingerich J., Emmenegger U., Eisen A., Zalewski P. (2013). Management of Carcinoma of the Penis: Consensus Statement from the Canadian Association of Genitourinary Medical Oncologists (CAGMO). Can. Urol. Assoc. J..

[5] Coelho R.W.P., Pinho J.D., Moreno J.S., Garbis D.V.E.O., do Nascimento A.M.T., Larges J.S., Calixto J.R.R., Ramalho L.N.Z., da Silva A.A.M., Nogueira L.R. (2018). Penile Cancer in Maranhão, Northeast Brazil: The Highest Incidence Globally?. BMC Urol..

[6] Czajkowski M., Falis M., Błaczkowska A., Rybarczyk A., Wierzbicki P.M., Gondek J., Matuszewski M., Hakenberg O.W. (2025). Penile Cancer Profile in a Central European Context: Clinical Characteristics, Prognosis, and Outcomes—Insights from a Polish Tertiary Medical Center. Cancers.

[7] Huang J., Chan S.C., Pang W.S., Liu X., Zhang L., Lucero-Prisno D.E., Xu W., Zheng Z.-J., Ng A.C.-F., Necchi A. (2024). Incidence, Risk Factors, and Temporal Trends of Penile Cancer: A Global Population-Based Study. BJU Int..

[8] Bray F., Ferlay J., Soerjomataram I., Siegel R.L., Torre L.A., Jemal A. (2018). Global Cancer Statistics 2018: GLOBOCAN Estimates of Incidence and Mortality Worldwide for 36 Cancers in 185 Countries. CA Cancer J. Clin..

[9] Wnętrzak I., Czajkowski M., Barańska K., Miklewska M., Wojciechowska U., Sosnowski R., Didkowska J.A. (2024). Epidemiology of Penile Cancer in Poland Compared to Other European Countries. Cancer Med..

[10] Morris B.J., Wamai R.G., Henebeng E.B., Tobian A.A., Klausner J.D., Banerjee J., Hankins C.A. (2016). Estimation of Country-Specific and Global Prevalence of Male Circumcision. Popul. Health Metr..

[11] European Association of Urology (2026). EAU-ASCO Guidelines on Penile Cancer.

[12] Mosquera Angulo H., Nieva-Posso D.A., García-Perdomo H.A. (2024). Sexuality in Penile Cancer Survivors: A Rarely Discussed Problem in Uro-Oncology. Int. J. Urol. Nurs..

[13] Anderson E., Yao H.H., Chee J. (2021). Optimal Surgical Margin for Penile-sparing Surgery in Management of Penile Cancer—Is 2 cm Really Necessary?. BJUI Compass.

[14] Hakenberg O.W., Compérat E.M., Minhas S., Necchi A., Protzel C., Watkin N. (2015). EAU Guidelines on Penile Cancer: 2014 Update. Eur. Urol..

[15] Brouwer O.R., Albersen M., Parnham A., Protzel C., Pettaway C.A., Ayres B., Antunes-Lopes T., Barreto L., Campi R., Crook J. (2023). European Association of Urology-American Society of Clinical Oncology Collaborative Guideline on Penile Cancer: 2023 Update. Eur. Urol..

[16] Roussel E., Peeters E., Vanthoor J., Bozzini G., Muneer A., Ayres B., Sri D., Watkin N., Bhattar R., Parnham A. (2021). Predictors of Local Recurrence and Its Impact on Survival after Glansectomy for Penile Cancer: Time to Challenge the Dogma?. BJU Int..

[17] Jazayeri S.B., Fazili A., Sirard R.B., Zemp L., Johnstone P.A.S., Chahoud J., Spiess P.E. (2025). Outcomes of PeIN at the Surgical Margin: Insights from an Organ-Sparing Penile Cancer Database. World J. Urol..

[18] WHO Classification of Tumours Editorial Board (2022). Urinary and Male Genital Tumours. WHO Classification of Tumours.

[19] Brierley J.D., Gospodarowicz M.K., Wittekind C. (2017). TNM Classification of Malignant Tumours.

[20] O’Connell K.A., Thomas J.L., Murad F., Zhou G., Sonpavde G.P., Mossanen M., Clinton T.N., Ji-Xu A., Alton K., Spiess P.E. (2024). Total Margin Control Is Superior to Traditional Margin Assessment for Treatment of Low-Stage Penile Squamous Cell Carcinoma. J. Urol..

[21] Danakas A.M., Bsirini C., Miyamoto H. (2018). The Impact of Routine Frozen Section Assessment During Penectomy on Surgical Margin Status and Long-Term Oncologic Outcomes. Pathol. Oncol. Res..

[22] Pang K.H., Yunis M., Haider A., Freeman A., Hadway P., Nigam R., Rees R., Muneer A., Alnajjar H.M. (2024). Outcomes of Intraoperative Frozen Section Examination of Surgical Resection Margins of the Penis in Penile Cancer. Clin. Genitourin. Cancer.

[23] Parnham A.S., Albersen M., Sahdev V., Christodoulidou M., Nigam R., Malone P., Freeman A., Muneer A. (2018). Glansectomy and Split-Thickness Skin Graft for Penile Cancer. Eur. Urol..

[24] Li J., Zhu Y., Zhang S.-L., Wang C.-F., Yao X.-D., Dai B., Ye D.-W. (2011). Organ-Sparing Surgery for Penile Cancer: Complications and Outcomes. Urology.

[25] Hoffman M.A., Renshaw A.A., Loughlin K.R. (1999). Squamous Cell Carcinoma of the Penis and Microscopic Pathologic Margins. Cancer.

[26] Agrawal A., Pai D., Ananthakrishnan N., Smile S.R., Ratnakar C. (2000). The Histological Extent of the Local Spread of Carcinoma of the Penis and Its Therapeutic Implications. BJU Int..

[27] Minhas S., Kayes O., Hegarty P., Kumar P., Freeman A., Ralph D. (2005). What Surgical Resection Margins Are Required to Achieve Oncological Control in Men with Primary Penile Cancer?. BJU Int..

[28] Philippou P., Shabbir M., Malone P., Nigam R., Muneer A., Ralph D.J., Minhas S. (2012). Conservative Surgery for Squamous Cell Carcinoma of the Penis: Resection Margins and Long-Term Oncological Control. J. Urol..

[29] Djajadiningrat R.S., van Werkhoven E., Meinhardt W., van Rhijn B.W.G., Bex A., van der Poel H.G., Horenblas S. (2014). Penile Sparing Surgery for Penile Cancer-Does It Affect Survival?. J. Urol..

[30] Cilio S., Tufano A., Pezone G., Alvino P., Spena G., Pandolfo S.D., Del Prete P., Amato C., Damiano R., Salonia A. (2023). Sexual Outcomes after Conservative Management for Patients with Localized Penile Cancer. Curr. Oncol..

[31] Sri D., Sujenthiran A., Lam W., Minter J., Tinwell B.E., Corbishley C.M., Yap T., Sharma D.M., Ayres B.E., Watkin N.W. (2018). A Study into the Association between Local Recurrence Rates and Surgical Resection Margins in Organ-Sparing Surgery for Penile Squamous Cell Cancer. BJU Int..

[32] Elst L., Roussel E., Miletic M., De Vries H.-M., Verdijk R., Yan S., Alnajjar H., Perri D., Kaur A., Sirard R.B. (2026). Local Recurrence after Glans-Sparing Surgery: No Impact on Penile Cancer-Specific Survival. BJU Int..

[33] Gunia S., Koch S., Jain A., May M. (2014). Does the Width of the Surgical Margin of Safety or Premalignant Dermatoses at the Negative Surgical Margin Affect Outcome in Surgically Treated Penile Cancer?. J. Clin. Pathol..

[34] Cubilla A.L. (2009). The Role of Pathologic Prognostic Factors in Squamous Cell Carcinoma of the Penis. World J. Urol..

