# Supplementary figures and images for: Surgical Margin Status and Minimal Margin Width in Penile Squamous Cell Carcinoma: Local Recurrence and Survival Outcomes in a Single-Centre Cohort
Source: Cancers (Basel). 2026 May 9;18(10):1535. doi: 10.3390/cancers18101535 (PMC13205046; doi:10.3390/cancers18101535)

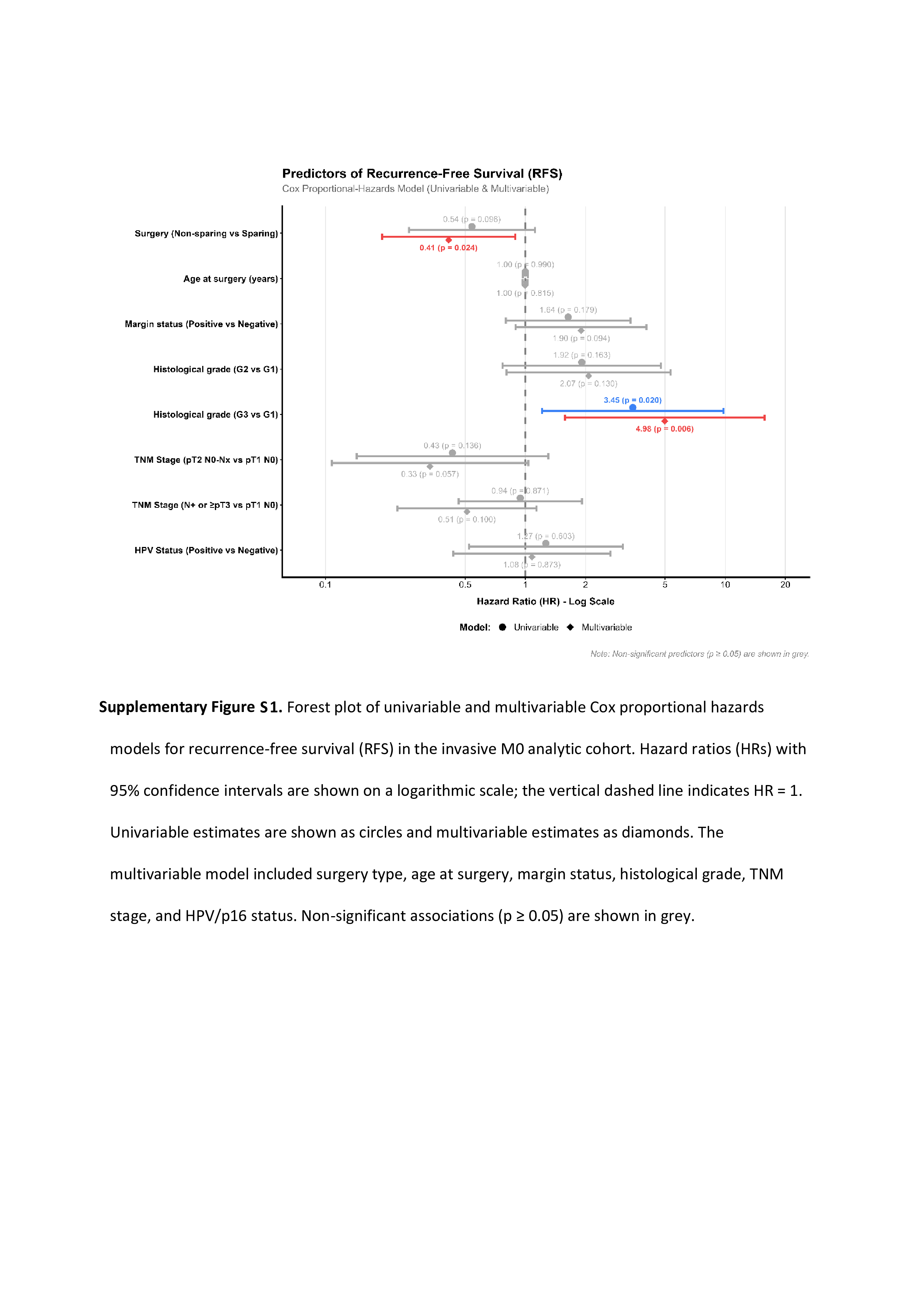

Supplement: Supplementary file 1 [file cancers-18-01535-s001.zip › Supplementary Figure S1.tiff]

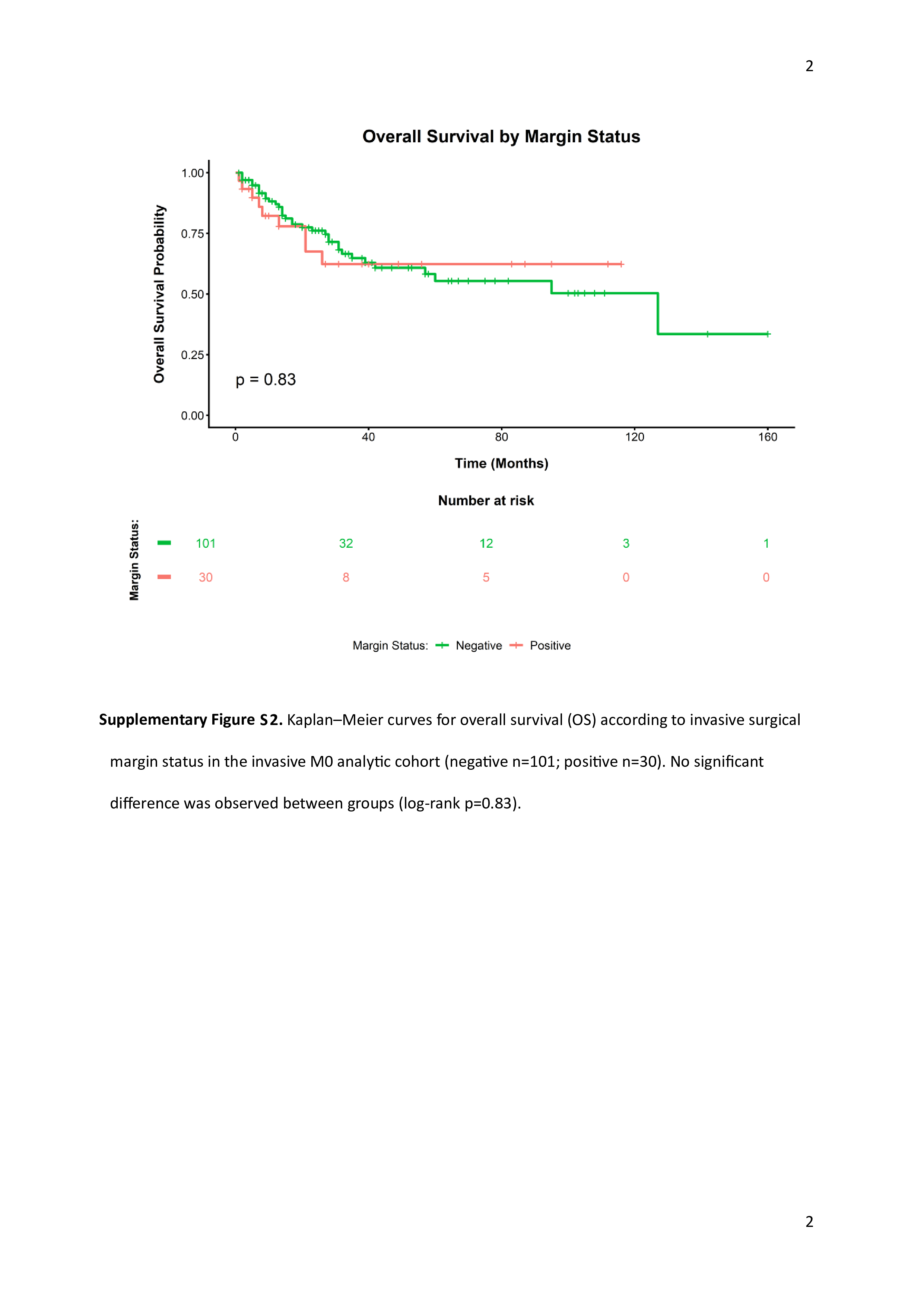

Supplement: Supplementary file 1 [file cancers-18-01535-s001.zip › Supplementary Figure S2.tiff]

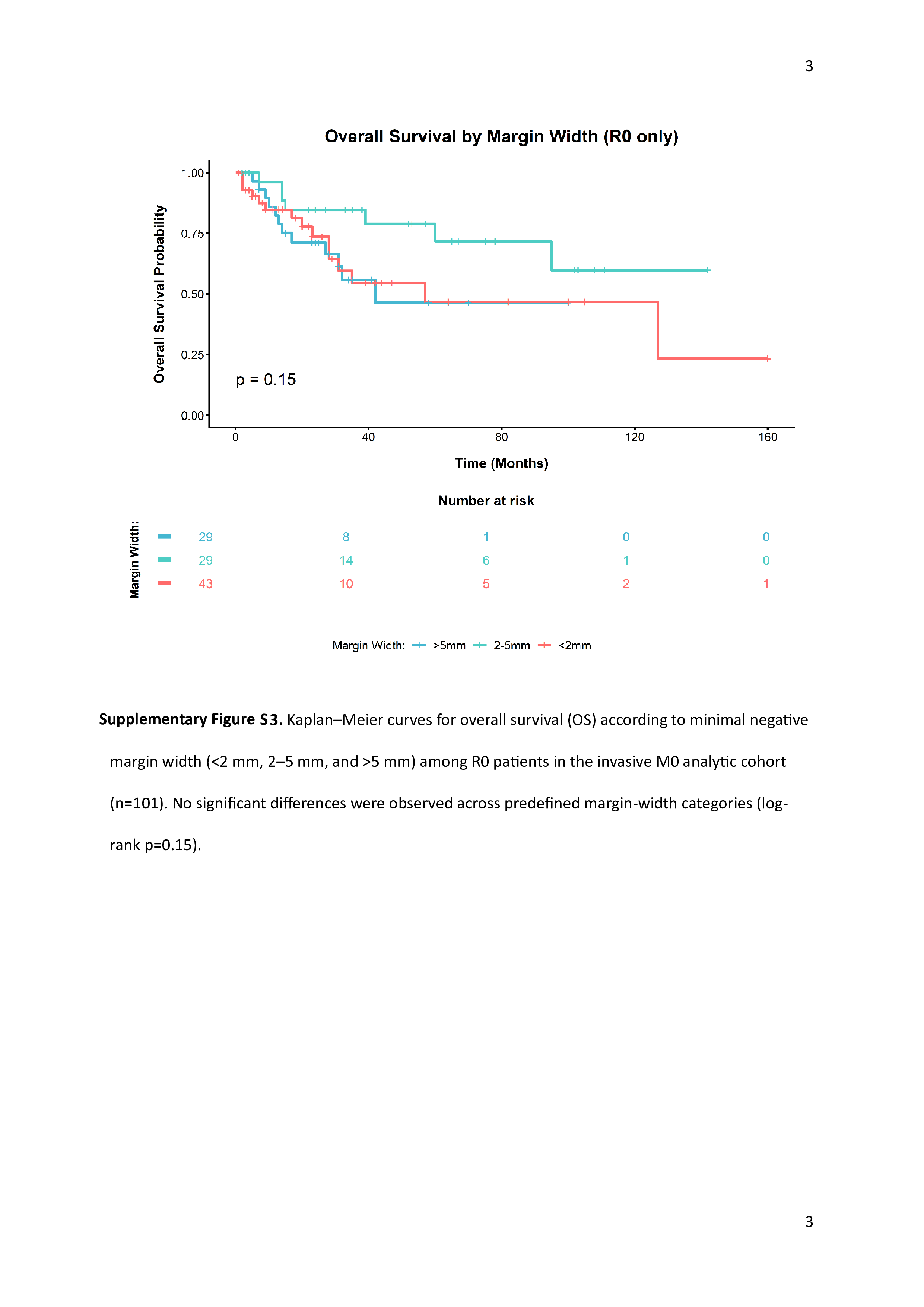

Supplement: Supplementary file 1 [file cancers-18-01535-s001.zip › Supplementary Figure S3.tiff]

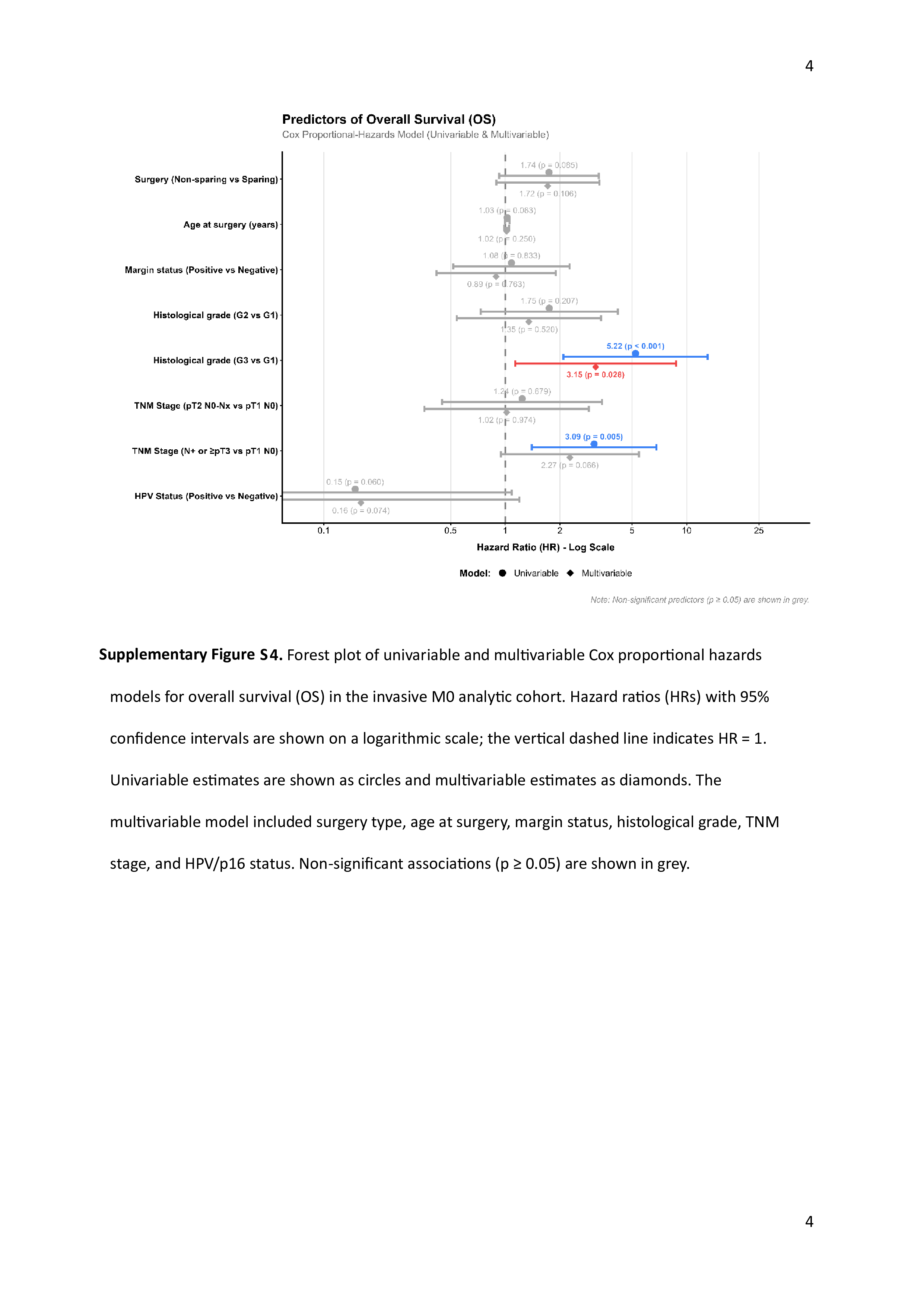

Supplement: Supplementary file 1 [file cancers-18-01535-s001.zip › Supplementary Figure S4.tiff]

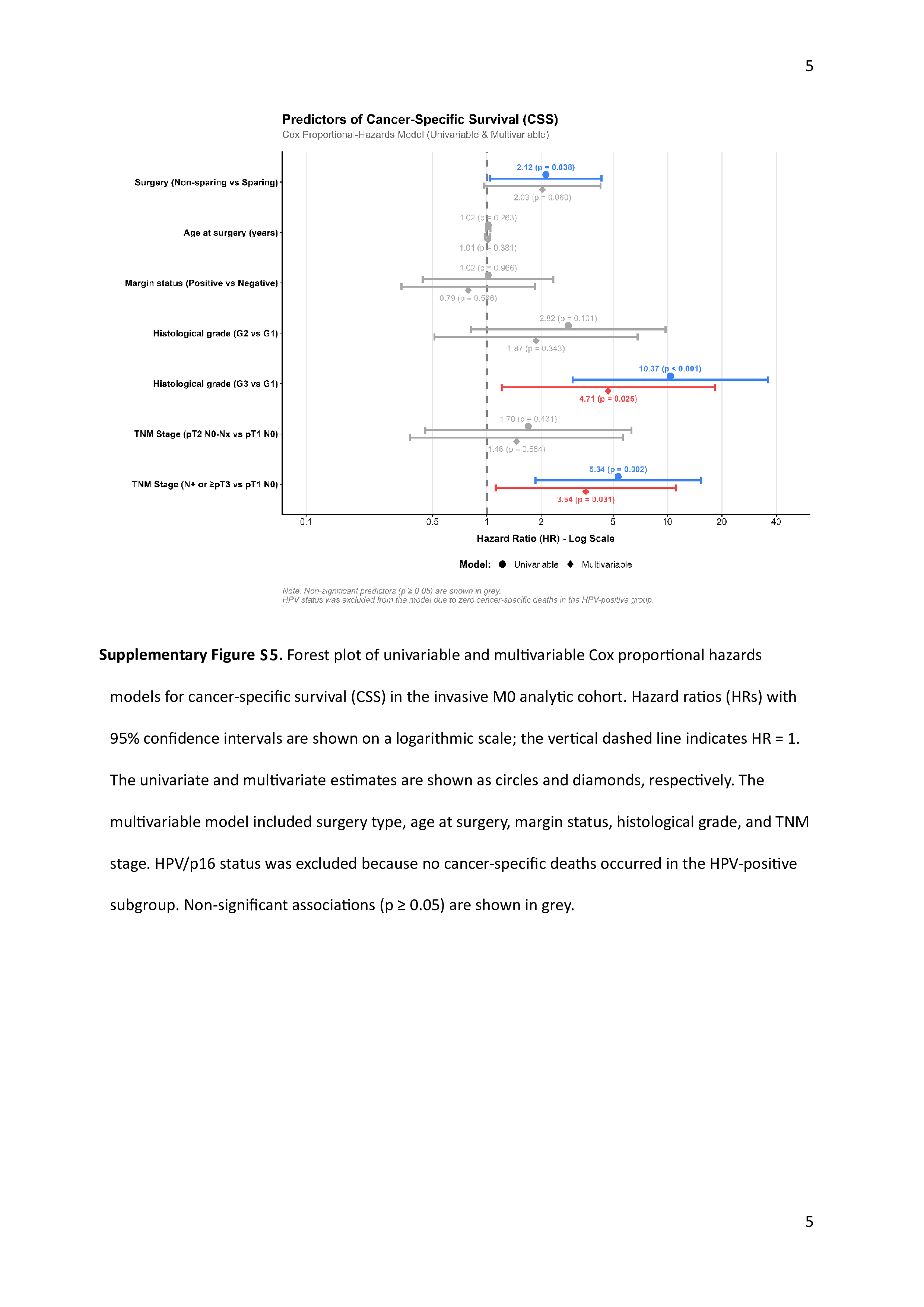

Supplement: Supplementary file 1 [file cancers-18-01535-s001.zip › Supplementary Figure S5.tiff]
